# Performance Evaluation of the Xpert® HCV Test on Fingerstick Blood in a Prospective Observational Clinical Study at CLIA-Waived Sites in the United States

**DOI:** 10.1093/cid/ciag173

**Published:** 2026-03-13

**Authors:** Jennifer R Havens, Shelly-Ann Fluker, Jonathan Schimmel, L Madeline McCrary, Lesley S Miller, Tomoko Udo, Yukari C Manabe, Anne Luetkemeyer, Greer Burkholder, Andrew M Moon, Cody A Chastain, Jennifer C Price, John Cafardi, Juan F Gallegos-Orozco, Brittany A Young, Jesse Young, Carlos Aparicio, Yu Song, Eric Lai, Gail E Louw

**Affiliations:** Department of Behavioral Science, University of Kentucky College of Medicine, Lexington, Kentucky, USA; Department of Medicine, Emory University School of Medicine, Atlanta, Georgia, USA; Department of Emergency Medicine, Mount Sinai Icahn School of Medicine, NewYork, New York, USA; Department of Medicine, Washington University School of Medicine, St.Louis, Missouri, USA; Department of Medicine, Emory University School of Medicine, Atlanta, Georgia, USA; Department of Health Policy, Management, and Behavior, College of Integrated Health Sciences, University at Albany, Albany, NewYork, USA; Department of Medicine, Johns Hopkins University School of Medicine, Baltimore, Maryland, USA; Department of Medicine, University of California SanFrancisco, San Francisco, California, USA; Department of Medicine, University of Alabama Birmingham Heersink School of Medicine, Birmingham, Alabama, USA; Department of Medicine, University of North Carolina School of Medicine, Chapel Hill, North Carolina, USA; Department of Medicine, Vanderbilt University Medical Center, Nashville, Tennessee, USA; Department of Medicine, University of SanFrancisco School of Medicine, San Francisco, California, USA; Department of Internal Medicine, The Christ Hospital, Cincinnati, Ohio, USA; Department of Internal Medicine, University of Utah School of Medicine, Salt Lake City, Utah, USA; Department of Internal Medicine, University of Utah School of Medicine, Salt Lake City, Utah, USA; Department of Pathology, University of Utah School of Medicine, Salt Lake City, Utah, USA; TriCore Reference Laboratories, Albuquerque, New Mexico, USA; CLAS Automation, Inc., Miami, Florida, USA; Cepheid, Sunnyvale, California, USA; PharmaDx, LLC, San Diego, California, USA; Cepheid, Sunnyvale, California, USA

**Keywords:** hepatitis C, viral RNA, point-of-care

## Abstract

**Background:**

A major barrier to hepatitis C virus (HCV) elimination in the United States is the lack of a point-of-care test to confirm the presence of HCV RNA. The purpose of this clinical trial was to evaluate the performance of the Xpert® HCV test at CLIA-waived sites in the United States.

**Methods:**

Participants at risk and/or with signs/symptoms of HCV infection provided fingerstick blood that was tested on the Xpert® HCV test and venous blood tested using the cobas® HCV and Elecsys® Anti-HCV II tests. Fingerstick blood was collected at CLIA-waived sites by individuals self-trained on collection procedures.

**Results:**

Participants (N = 1279) were enrolled across 15 sites; 1015 (79.3%) were deemed eligible for further evaluation. Specimens from 985 (97.0%) participants with valid results for Xpert®, cobas and Elecsys were included in the performance analysis. The prevalence of HCV antibodies and HCV RNA was 34.6% and 12.4%, respectively. The Xpert® HCV test demonstrated a positive percent agreement of 93.4% (95% CI: 87.6–96.6) and a negative percent agreement of 99.8% (95% CI: 99.2–99.9) relative to the patient infected status.

**Conclusions:**

Data from this clinical trial showed that the Xpert® HCV test was sensitive, specific, and acceptable for use to detect HCV RNA in human EDTA fingerstick blood from individuals at risk and/or with signs/symptoms of HCV infection.

***Clinical Trials Registration.*** This study, Pro0075996, was approved by Advarra IRB (Columbia, Maryland, 21044) and registered on ClinicalTrials.gov (NCT06508996).

## Teaser

This study evaluated the clinical performance of Xpert® HCV on fingerstick blood at CLIA-waived sites in the United States. Results demonstrated that Xpert® HCV showed good ability to detect HCV RNA in individuals with active HCV infection.

**(See the Editorial Commentary by Furukawa et al on pages e79–80.)**

Hepatitis C virus (HCV) infection impacts tens of millions of people worldwide [1] with 1 million incident infections annually [2, 3]. As the first step in the care cascade, screening and diagnosis of HCV are key to elimination; however, there is considerable loss to follow-up at the screening and diagnosis steps [4]. A major barrier to streamlined diagnosis and treatment in the United States is the lack of a point-of-care (POC) test to establish the presence of HCV RNA. POC RNA tests, where results are available within an hour, are approved for the diagnosis of acute and chronic HCV infections in other countries across the globe [5] and offer several advantages. For example, POC testing can be utilized in CLIA-waived settings, thereby greatly expanding the locations in which screening can effectively occur and the number of individuals treated. Additionally, POC testing is associated with improved time to treatment initiation [6, 7].

The development of the Xpert® HCV VL Fingerstick paved the way for the Xpert® HCV test [5, 8], and the Xpert® HCV VL Fingerstick was one of the first molecular POC HCV RNA tests to receive CE marking [9], with extensive studies confirming its clinical performance [10, 11]. Other POC HCV RNA tests have also been evaluated, but uptake has been limited largely due to a more complicated workflow [11, 12]. There are several notable differences between the Xpert® HCV VL Fingerstick and Xpert® HCV tests. Xpert® HCV is designed to be simpler to perform and is a qualitative test that can be run by untrained users in CLIA-waived settings. The Xpert® HCV test also has an early termination feature, whereby results are available in ∼40 minutes for HCV RNA-detectable samples and 60 minutes for HCV RNA-undetectable samples. Another notable difference is the sample collection; 250–500 µL are collected into a BD Microtainer® collection tube, 100 µL of which is pipetted into the Xpert® HCV cartridge. This difference in fingerstick collection procedures was required since the Minivette® POCT used in sample collection for the Xpert® HCV VL Fingerstick is not FDA-cleared.

The purpose of this study was to evaluate the clinical performance of the Xpert® HCV test in K2-EDTA fingerstick blood collected from symptomatic and/or individuals at risk of HCV infection when performed by untrained users on the GeneXpert® Xpress System in a CLIA-waived setting.

## METHODS

### Study Design and Setting

**Figure 1.**
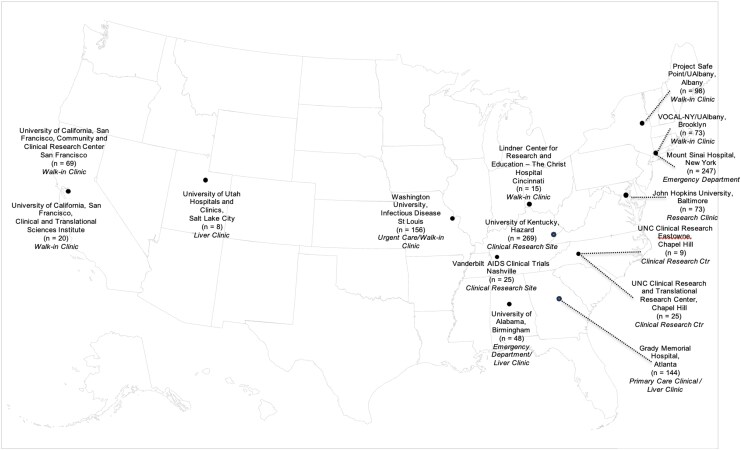
Participant enrollment by Xpert® HCV testing site locations and site type (N & 1279). Abbreviation: HCV, hepatitis C virus.

We conducted a multicenter, prospective clinical study at 15 geographically diverse locations in the United States (Figure 1). These 15 CLIA-waived sites (eg primary care clinics, urgent care, emergency departments, and clinical research centers) participated in the prospective enrollment of participants between 6 February 2024 and 10 May 2024. This study aimed to prospectively collect fingerstick blood from 1000 participants, in order to meet the sample size requirements of 120 HCV positive (by patient infected status [PIS] classification) and 500 HCV Negative (by PIS) individuals, assuming a 12% prevalence of HCV infection (by PIS) in the study population.

All sites conducted paired collection of fingerstick and venous whole blood and subsequent testing by untrained users of the fingerstick blood on the Xpert® HCV test using the GeneXpert® Xpress system. Fingerstick blood was collected by individuals self-trained on the collection procedures according to the Xpert® HCV investigational use only instructions for use, manufacturer's instructions, and fingerstick collection tutorial video.

A reference laboratory (Tricore Research Institute, Albuquerque, NM, USA) conducted testing on the cobas® HCV test (Roche Diagnostics, Indianapolis, Indiana, USA) and the Elecsys® Anti-HCV II test (Roche Diagnostics, Indianapolis, Indiana, USA) using serum obtained from the venous whole blood specimens. Institutional review board (IRB) approval of the clinical study protocol for all specimen collection sites was obtained through Advarra IRB (Columbia, Maryland, 21044). Consent was obtained prior to the initiation of any study procedures and the trial was registered on ClinicalTrials.gov (NCT06508996). Participants were remunerated for study participation in the form of cash, gift card, or check.

### Participant Eligibility

**Figure 2.**
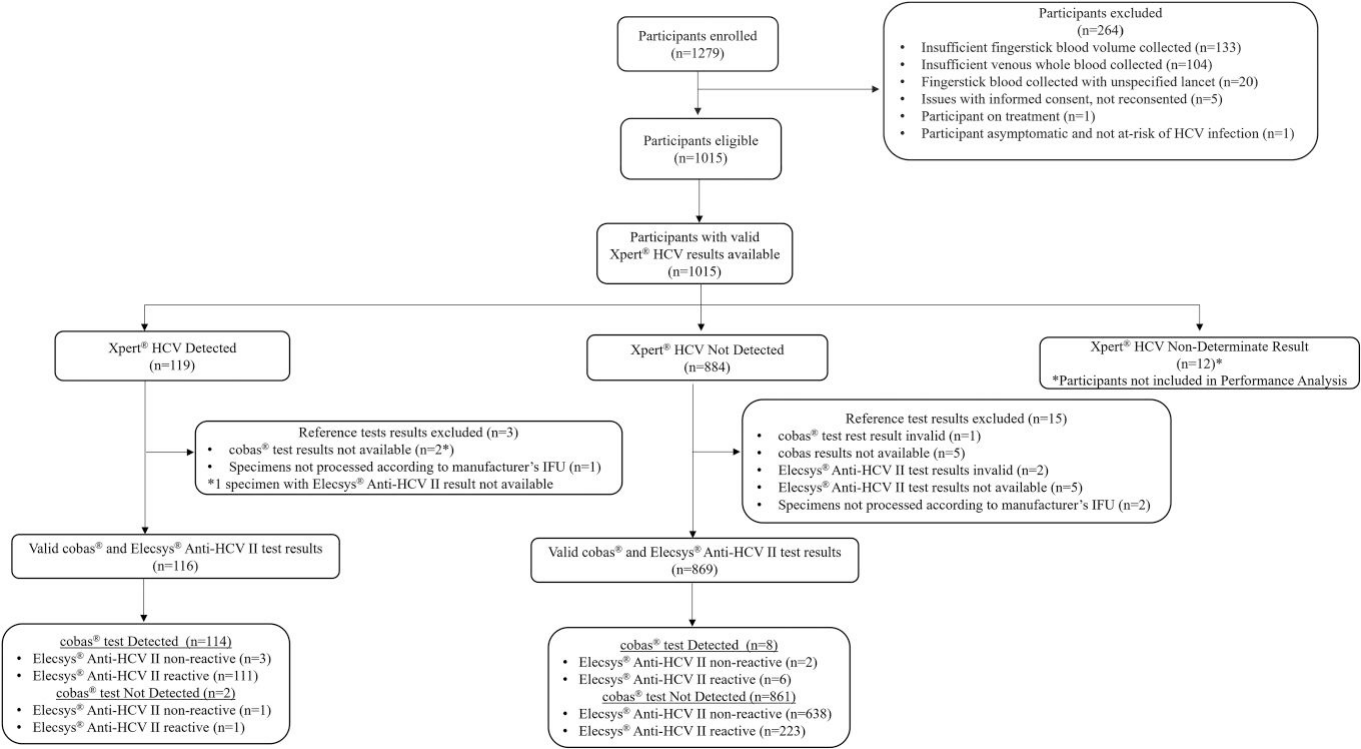
Participant flow diagram.

The participant flow diagram for the study is presented in Figure 2 following STARD guidelines [13]. Study participants were individuals ≥18 years or older who met the following inclusion criteria: (1) provided documented informed consent, (2) were not on treatment for HCV infection at the time of enrollment based on review of medical records or self-report, (3) were able to and agreed to provide 1× ≥ 250 µL fingerstick blood in BD microtainer and 2× 5 mL venous whole blood in serum separator tubes (SST) and, (4) were considered at risk and/or showed signs/symptoms of HCV infection as defined in CDC [14] and WHO [15] guidelines.

If available, the medical records of participants were reviewed. A brief questionnaire was also administered to consenting participants to ascertain age, sex, race/ethnicity, lifetime injection drug use, self-reported HCV risk factors and symptoms, and prior history of HCV infection.

### Specimen Collection

Fingerstick blood (1 tube of ≥250 µL) was collected using BD Microtainer® Contact-Activated Lancet (366594) in K2-EDTA BD Microtainer® blood collection tubes (365974) according to the manufacturer's (Becton, Dickinson and Company) instructions, and venous whole blood (VWB) (2 tubes of ≥5 mL each) was collected by venipuncture in SST (gold top).

### Xpert® HCV Test

The Xpert® HCV test (Cepheid, Sunnyvale, California, USA), performed on the GeneXpert® Xpress System (Cepheid, Sunnyvale, California, USA), is an automated in vitro reverse transcription polymerase chain reaction (RT-PCR) test for the qualitative detection of HCV RNA in human fingerstick K2-EDTA whole blood from adult individuals at risk and/or with signs/symptoms for HCV infection with or without antibodies to HCV. Detection of HCV RNA indicates that the virus is replicating and therefore is evidence of active infection. Detection of HCV RNA does not discriminate between acute and chronic states of infection. The LoD of the Xpert HCV test was previously independently determined according to CLSI EP-17 A2, by testing dilutions of the 6th WHO International Standard (NIBSC Code: 18/184) for HCV RNA for genotypes 1a and clinical isolates for genotypes 1b, 2b, 3a, 4, 5, and 6 in HCV negative human fingerstick whole blood. Probit analysis demonstrated LoD range of 35.0 to 136.4 IU/mL, depending on the genotype [16, 17].

### Test Procedures

#### Xpert® HCV

After collecting 250 µL fingerstick blood in the BD Microtainer, a volume of 100 µL fingerstick blood was transferred to the Xpert® HCV test cartridge within 60 minutes of collection, using the 100 µL transfer pipette included in the test kit. The Xpert® HCV cartridge was subsequently loaded into the GeneXpert® Xpress System. A “NO-RESULT—REPEAT TEST” may be due to specimen volume, probe check or internal control issues, while an “INSTRUMENT ERROR” may be due to exceeding the maximum pressure limit or an instrument component failure. If a “NO RESULT—REPEAT TEST” or “INSTRUMENT ERROR” result was obtained by fingerstick blood testing, the testing was repeated once using blood from the same sample collection tube, according to the manufacturer's instructions. If the retest result was also non-determinant, the test result was reported as such.

Venous whole blood specimens were drawn and stored at −20°C until shipped to the central laboratory for processing. Comparator testing was conducted using the cobas® HCV test (Roche Diagnostics, Indianapolis, Indiana, USA) and the Elecsys® Anti-HCV II test (Roche Diagnostics, Indianapolis, Indiana, USA) on serum samples according to manufacturer's instructions.

### Performance Evaluation

The performance of the Xpert® HCV test on fingerstick blood was compared with the PIS, which consisted of results from both the cobas® HCV and Elecsys® Anti-HCV II test. PIS was categorized as active chronic infection (HCV antibody reactive, HCV RNA detected), past/resolved infection (HCV antibody reactive, HCV RNA not detected), active acute infection (HCV antibody non-reactive, HCV RNA detected), and not infected (HCV antibody non-reactive, HCV RNA not detected).

### Statistical Analysis

The initial and final non-determinant proportions for the Xpert® HCV test were calculated as a percent of the number of specimens with non-determinant results out of the total number of eligible specimens tested. Discrepant results between the Xpert® HCV test and the cobas® HCV test were further evaluated by investigating the assay files, specimen handling procedures, shipping and storage conditions, adherence to testing procedures, and available clinical/laboratory information. Positive and negative percent agreement (NPA) were determined for the Xpert® HCV test relative to PIS that included results from both the cobas® test and the Elecsys® Anti-HCV II test. Data were analyzed using SAS software, Version 9.4 (Cary, North Carolina, USA).

## RESULTS

### Participants Characteristics

One thousand two hundred seventy-nine participants were enrolled during the study period. Of these, 264 participants were deemed ineligible for inclusion in the final study population due to (1) insufficient fingerstick blood collected (n=133); (2) insufficient VWB collected (n=104); (3) fingerstick blood collected with unspecified lancet (n=20); (4) issue with consent, not reconsented (n= 5); (5) participant on treatment (n=1); and (6) asymptomatic/not at risk for HCV infection) (n=1) (Figure 2). A total of 1015 specimens were eligible to be included in the study. Additionally, 18 specimens were excluded due to the following: (1) reference test(s) results not available (n = 12), (2) reference test(s) results invalid (n = 3), and (3) specimens not processed according to manufacturer's instructions (n = 3). Moreover, 12 specimens were excluded due to unresolved repeat non-determinant result by the Xpert® HCV test. Therefore, 985 specimens were deemed suitable for inclusion in the performance assessment.

The demographic and clinical characteristics for the 1015 participants deemed eligible for inclusion are shown in Table 1. The majority of the participants were between 22 and 60 years of age, male, and 43.1% had a history of injection drug use.

**Table 1.** Demographic and Clinical Characteristics of Eligible Participants (N = 1015)

Demographic and Clinical Characteristics		Overall (N = 1015)
Age	> = 18 y old <22	3 (0.3%)
	> = 22 y old ≤60	646 (63.6%)
	>60	366 (36.1%)
Gender	Male	546 (53.8%)
	Female	469 (46.2%)
Race^a^	White	522 (51.4%)
	Black/African American	371 (36.6%)
	Other^b^	93 (9.2%)
	Asian	10 (1.0%)
	Unknown/prefer not to answer	18 (1.8%)
	Missing	1 (0.1%)
Ethnicity	Hispanic/Latino	130 (12.8%)
	Not Hispanic/Latino	865 (85.2%)
	Unknown/prefer not to answer	16 (1.6%)
	Missing	4 (0.4%)
History of HCV infection	Yes	293 (28.9%)
	No	720 (70.9%)
	Data not available	2 (0.2%)
Standard of care HCV antibody test	Yes	547 (53.9%)
	Not available	158 (15.6%)
	Never had an HCV antibody test	308 (30.3%)
	Missing	2 (0.2%)
Results of recent HCV antibody test	Reactive	164 (16.2%)
	Not reactive	379 (37.3%)
	Invalid	3 (0.3%)
	No result^c^	469 (46.2%)
Recent HCV NAAT test	Yes	204 (20.1%)
	Not available	196 (19.3%)
	Never had an HCV NAAT test	614 (60.5%)
	Missing	1 (0.1%)
Result of recent HCV NAAT test	Positive	34 (3.3%)
	Negative	170 (16.7%)
	No result^d^	811 (79.9%)
HCV genotype test	Yes	46 (4.5%)
	Not available	246 (24.2%)
	Never had an HCV genotyping test	722 (71.1%)
	Missing	1 (0.1%)
Genotype test result	1a	30 (3.0%)
	1b	6 (0.6%)
	1c	2 (0.2%)
	2b	2 (0.2%)
	3a	5 (0.5%)
	No result^e^	970 (95.6%)
Treatment history	Ever treated	175 (17.2%)
	Never been treated	839 (82.7%)
	Missing	1 (0.1%)
Symptomatic	Yes	374 (36.8%)
	No	641 (63.2%)
At risk	Yes	936 (92.2%)
	No	79 (7.8%)
Symptomatic and at risk	Yes	295 (29.1%)
	No	720 (70.9%)
History of injection drug use	Yes	437 (43.1%)
	No	578 (56.9%)
History of non-HCV liver disease^f^	Yes	68 (6.7%)
	No	946 (93.2%)
	Missing	1 (0.1%)
HIV status	Positive	156 (15.4%)
	Negative	800 (78.8%)
	Unknown (never tested)	59 (5.8%)
HBV status	Positive	16 (1.6%)
	Negative	687 (67.7%)
	Unknown (never tested)	311 (30.6%)
	Missing	1 (0.1%)

^a^If more than one race is reported for a participant, they are only captured in one category.

^b^Other race group includes “American-Indian or Alaskan Native,” “More than one race,” “Native Hawaiian or Pacific Islander,” and “Other.”

^c^Combined category of “Not Available,” “Never had a HCV Antibody Test,” and “Missing.”

^d^A combined category of “Not Available,” “Never had a HCV NAAT Test,” and “Missing.”

^e^A combined category of “Not Available,” Never had a HCV Genotyping Test,” and “Missing.”

^f^Includes fatty liver disease, metabolic dysfunction-associated steatotic liver disease (MASLD), primary biliary cirrhosis, chronic HBV, alcohol-associated liver disease, autoimmune hepatitis, and other non-HCV liver disease.

### Performance of Xpert® HCV Test Relative to PIS

The Xpert® HCV test demonstrated a positive percent agreement (PPA) of 93.4% (95% CI: 87.6–96.6) and an NPA of 99.8% (95% CI: 99.2–99.9) relative to PIS and the prevalence of HCV RNA-detectable specimens was 12.4% (122/985) (Table 2). The Xpert® HCV test was able to detect HCV RNA in 111 (94.9%) specimens in the active chronic infection group and 3 (60.0%) in the active acute infection group. In addition, the Xpert® HCV test did not detect HCV RNA in 223 (99.5%) specimens in the past/resolved infection group and 638 (99.8%) in the not infected group (Supplementary Table 1).

**Table 2.** Performance of Xpert® HCV Test Results Relative to PIS

		Patient Infected Status		
		HCV Positive^a^	HCV Negative^b^	Total
Xpert® HCV test	HCV detected	114	2	116
	HCV not detected	8	861	869
	Total	122	863	985
PPA (95% CI)		93.4% (95% CI: 87.6–96.6)		
NPA (95% CI)		99.8% (95% CI: 99.2–99.9)		
Prevalence		12.4%		

^a^Active chronic or acute infection.

^b^Past/resolved infection or not infected.

There were 10 specimens with discrepant results with the clinical and laboratory information detailed in Supplementary Table 2. In addition, upon initial testing, 6.0% of specimens resulted in non-determinant results (61/1015), with retesting resulting in 1.2% (12/1015) non-determinant results.

The Xpert® HCV test demonstrated clinical performance relative to the PIS in HCV antibody reactive specimens with a PPA of 94.9% (95% CI: 89.3–97.6) in the active chronic infection group and an NPA of 99.6% (95% CI: 97.5–99.9) in the past/resolved infection group and the overall HCV antibody prevalence was 34.6% (Table 3).

**Table 3.** Performance of Xpert® HCV Test Results Relative to cobas® HCV Test Results in HCV Antibody Reactive Specimens

		cobas® HCV Test		
		HCV Positive	HCV Negative	Total
Xpert® HCV test	HCV detected	111	1	112
	HCV not detected	6	223	229
	Total	117	224	341
PPA		94.9% (95% CI: 89.3–97.6)		
NPA		99.6% (95% CI: 97.5–99.9)		

The clinical performance Xpert® HCV test relative to the PIS in HCV antibody non-reactive specimens showed a PPA of 60.0% (95% CI: 23.1–88.2) in the active acute infection and an NPA of 99.8% (95% CI: 99.1–100.0) in the not infected study population (Table 4).

**Table 4.** Performance of Xpert® HCV Test Results Relative to cobas® HCV Test Results in HCV Antibody Non-Reactive Specimens

		cobas® HCV Test		
		HCV Positive	HCV Negative	Total
Xpert® HCV test	HCV detected	3	1	4
	HCV not detected	2	638	640
	Total	5	639	644
PPA		60.0% (95% CI: 23.1–88.2)		
NPA		99.8% (95% CI: 99.1–100.0)		

## DISCUSSION

This multicenter, prospective clinical study of 985 participants at 15 CLIA-waived sites across the US demonstrated good performance of the Xpert® HCV test relative to PIS (PPA of 93.4%; NPA of 99.8%). The performance of the Xpert® HCV test was consistent with studies evaluating the molecular performance of the Xpert® HCV VL Fingerstick [5]. A systematic review of the diagnostic accuracy of the Xpert® HCV VL fingerstick test across 7 studies had a sensitivity and specificity for HCV RNA detection of 99% (95% CI: 97%–99%) and 99% (95% CI: 94%–100%), respectively [10]. Additionally, a systematic review of the diagnostic accuracy of POC HCV RNA assays for diagnosis of HCV demonstrated a pooled sensitivity of 99% (95% CI: 98%–99%) and specificity of 99% (95% CI: 99%–100%) [11]. The NPA of 99.8% in our study is similar to these studies but the PPA of 93.4% in our study is lower. This could be due to slight differences in the LoD of the qualitative Xpert® HCV test and the quantitative Xpert® HCV VL fingerstick test, differences in the participant population in the study, differences in the collection device, and/or differences in the comparator methods.

The FDA reviewed the results of this study under its de novo premarket review pathway and found that the Xpert® HCV test provides reasonable assurance of safety and effectiveness for the intended use. The FDA authorized the Xpert® HCV in June of 2024 [18]. This is the first HCV RNA test approved from fingerstick blood, without specimen processing required. In October 2024, the CDC published guidelines [19] for consideration of implementation for POC HCV RNA testing, highlighting that setting characteristics influence the HCV testing approach with the following settings favoring POC RNA testing: high HCV prevalence, brief encounter settings, and settings with no phlebotomy or laboratory access. The CDC and AASLD/IDSA also emphasized the benefit of POC testing is maximized when paired with highly accessible HCV treatment [17, 20, 21].

Several characteristics of the Xpert® HCV test make it useful in these settings. Venous blood access can be challenging among many individuals, and many settings serving high-risk individuals do not have access to phlebotomy. The Xpert® HCV test uses a fingerstick sample and ∼10% of participants were excluded due to insufficient fingerstick sample. The proportion of insufficient sample collection in this study of Xpert® HCV is higher than studies of the Xpert® HCV VL fingerstick [5, 8], which is likely due to differences in sample collection procedures with the microtainer stipulating minimum collection of 250 µL (compared with 100 µL). For this study, inadequate sample collection was more frequent at 2 of the 15 sites accounting for ∼40% of this sample set. Within the 2 sites, a single untrained self-trained user was responsible for many (∼39.1%) of the samples with insufficient volume. It should be noted that sufficient sample collection often improves over time with sufficient training and experience [22]. Subsequent to study completion, fingerstick collection training materials in addition to the manufacturer's instructions have been updated to aid in sample collection.

A prospective study done on the Xpert® HCV test prior to the clinical trial established the sample stability for fingerstick blood of 4 hours, which enables retesting from the same sample collected if initial testing produces in a non-determinant result [23]. Of note, the final proportion of non-determinant results of 1.2% was low, consistent with prior studies [8], increasing the utility of the test in these settings.

There are limitations to the Xpert® HCV test. There is a potential for false positive results due to contamination during sample collection, however, due to the closed cartridge design of the Xpert® HCV test, the possibility of amplicon contamination is very low. Additionally, there is the potential for false negative results, which may occur when very low levels of HCV RNA are not detected [24]. In this study, investigation into discrepant results did not provide sufficient evidence to ascertain a root cause; however, differences in the LoD between the qualitative Xpert® HCV test (35 to 135 IU/mL) [23] in fingerstick blood and the quantitative cobas® HCV test (13.7 IU/mL) in serum likely explains some false negative results. Additionally, the 4 specimens with the cobas detectable, not quantifiable results, is interpreted analytically within the manufacturer's instructions as “HCV RNA detected but not quantified.” This may demonstrate low-level viremia, which could be indicative of previous infection with limited current clinical relevance.

The GeneXpert® Xpress System has limited capacity for high volume testing, as the device runs up to 4 samples simultaneously. Additionally, depending on the setting, the price of the instrument and ongoing service may make testing with the Xpert® HCV more expensive than other diagnostic strategies. There is data, however, supporting cost effectiveness of a one-step HCV RNA POC strategy in high-risk populations. A study utilizing a health economic model to evaluate the cost and outcomes of a one-step HCV RNA POC diagnostic strategy compared with SoC among PWID in the Medicaid population, SoC suggested that a POC testing strategy could yield improved outcomes at lower cost in a high-risk population [25]. Additional studies also suggest that, compared with SoC, one-step HCV RNA POC diagnosis may be cost effective [26–28] However, studies that have evaluated cost effectiveness of strategies for diagnosing HCV infection in other countries found that a two-step POC-based strategy (POC HCV antibody followed by either POC or dried blood spot-based HCV RNA testing) may be most effective [26–28]. The limitations of the HCV Xpert® test were taken into account in the 2024 CDC guidance on implementation of HCV RNA POC testing, which describes settings that favor POC HCV RNA and describes approaches to POC HCV RNA instrument and cartridge procurement [19].

In conclusion, this study demonstrated that the Xpert® HCV test has the ability to detect HCV RNA in individuals with active HCV infection, regardless of HCV antibody status. Data for this study led to the FDA authorization for clinical use. This is a critically important step in HCV elimination in the US given the need for screening efforts to be coupled with building infrastructure to support testing, linkage to care, and treatment of infected individuals. The availability of POC HCV RNA testing has significant potential to improve treatment access and move the United States closer to elimination of HCV.

## Supplementary Material

ciag173_Supplementary_Data

## Supplementary Data

Supplementary materials are available at *Clinical Infectious Diseases* online. Consisting of data provided by the authors to benefit the reader, the posted materials are not copyedited and are the sole responsibility of the authors, so questions or comments should be addressed to the corresponding author.
