# Supplementary material for: Performance Evaluation of the Xpert® HCV Test on Fingerstick Blood in a Prospective Observational Clinical Study at CLIA-Waived Sites in the United States
Source: Clin Infect Dis. 2026 Mar 13;83(1):e71–8. doi: 10.1093/cid/ciag173 (PMC13320264; doi:10.1093/cid/ciag173)
Supplement: ciag173_Supplementary_Data [file ciag173_supplementary_data.zip › SupplementaryTable1.R2.docx]

**Supplementary Table 1. Xpert^®^ HCV Test Results relative to PIS (N=985)**

| **ELECSYS HCV Antibody** | **cobas HCV** | **PIS Classification** | **Xpert^®^ HCV** | **N (%)** |
| --- | --- | --- | --- | --- |
| Reactive | Detected^a^ | Active Chronic Infection | HCV DETECTED | 111 (11.3%) |
|  |  |  | HCV NOT DETECTED | 6 (0.6%) |
|  | Not detected^b^ | Past/resolved infection | HCV DETECTED | 1 (0.1%) |
|  |  |  | HCV NOT DETECTED | 223 (22.6%) |
| Non-reactive | Detected | Active Acute Infection | HCV DETECTED | 3 (0.3%) |
|  |  |  | HCV NOT DETECTED | 2 (0.2%) |
|  | Not detected | Not Infected | HCV DETECTED | 1 (0.1%) |
|  |  |  | HCV NOT DETECTED | 638 (64.8%) |
| **Total** | | | | 985 |
| ^a^ “HCV detected” result for the cobas**^®^** HCV test is reported as either: “< Titer Min”, “15 IU/mL≤ Titer < 25 IU/mL”, “25 IU/mL ≤ Titer ≤ Titer Max”, or “> Titer Max”. The analytical interpretation for “<Titer Min” is HCV RNA detected but not quantified. ^b^ “HCV not detected” result for the cobas**^®^** HCV test is reported as “Target Not Detected”. The analytical interpretation for “Target Not Detected” is HCV RNA not detected. | | | | |
