# Supplementary material for: Performance Evaluation of the Xpert® HCV Test on Fingerstick Blood in a Prospective Observational Clinical Study at CLIA-Waived Sites in the United States
Source: Clin Infect Dis. 2026 Mar 13;83(1):e71–8. doi: 10.1093/cid/ciag173 (PMC13320264; doi:10.1093/cid/ciag173)
Supplement: ciag173_Supplementary_Data [file ciag173_supplementary_data.zip › SupplementaryTable2.R2.docx]

**Supplementary Table 2: Clinical and Laboratory Information of Specimens with Discrepant Results**

| **Obs** | **Specimen ID** | **Discordance** | **Elecsys Result** | **Xpert**^®^ **Result** | **Xpert**^®^ **Ct** | **cobas^®^ HCV Test Results** | | **Baseline Clinical Information** | | |
| --- | --- | --- | --- | --- | --- | --- | --- | --- | --- | --- |
|  |  |  |  |  |  | **cobas^®^ HCV**  **Result** | **Viral Load (IU/ml)** | **Symptoms^a^** | **Risk Factor^b^** | **HCV Infection History** |
| 1 | 293C595242CWB^c^ | FP | NON-REACTIVE | HCV DETECTED | 23.9 | NEGATIVE | TND^d^ | 9 | 5 | Yes |
| 2 | 293C596B025CWB | FP | REACTIVE | HCV DETECTED | 41.1 | NEGATIVE | TND | 2, 4, 5, 8, 9 | 1, 5 | Yes |
| 3 | 293C013073CWB | FN | NON-REACTIVE | HCV NOT DETECTED | 0 | POSITIVE | 4.44e+01 | 2, 3, 5, 7, 8, 9 | N/A^e^ | No |
| 4 | 293C238130CWB | FN | NON-REACTIVE | HCV NOT DETECTED | 0 | POSITIVE | Detectable, not quantifiable | N/A^f^ | 2 | No |
| 5 | 293C013094CWB | FN | REACTIVE | HCV NOT DETECTED | 0 | POSITIVE | 1.14e+02 | 2, 5, 7, 8, 9 | 1 | Yes |
| 6 | 293C013198CWB | FN | REACTIVE | HCV NOT DETECTED | 0 | POSITIVE | Detectable, not quantifiable | 7, 8, 9 | 1 | Yes |
| 7 | 293C013219CWB | FN | REACTIVE | HCV NOT DETECTED | 0 | POSITIVE | 3.74e+02 | 2, 4, 6, 7, 8, 9 | 1 | Yes |
| 8 | 293C596A056CWB | FN | REACTIVE | HCV NOT DETECTED | 0 | POSITIVE | Detectable, not quantifiable | 2, 5, 7, 8 | 1, 5, 9 | Yes |
| 9 | 293C238016CWB | FN | REACTIVE | HCV NOT DETECTED | 0 | POSITIVE | Detectable, not quantifiable | N/A^f^ | 1, 5 | No |
| 10 | 293C595238CWB^c^ | FN | REACTIVE | HCV NOT DETECTED | 0 | POSITIVE | 2.22e+06 | N/A^f^ | 5 | No |
| ^a^ Symptoms include: 1) Jaundice; 2) Nausea or vomiting; 3) Fever; 4) Dark-colored urine; 5) Abdominal pain; 6) Light-colored stool; 7) Loss of appetite; 8) Fatigue; 9) Joint pain  ^b^ Risk Factor: 1) Intravenous drug use; 2) Persons who are HIV-positive; 3) Blood transfusion or organ transplant received prior to 1992; 4) Blood clotting factor for hemophilia received prior to 1987; 5) Born between 1945 and 1965; 6) Born to an HCV-infected mother; 7) Chronic hemodialysis patients; 8) Persons who engage in high-risk sexual behavior; 9) Persons with known exposure to HCV, such as healthcare workers after needle stick involving HCV-positive blood; recipients of blood organs from a donor who tested HCV-positive  ^c^ 293C595238CWB and 293C595242CWB: samples with suspicion of specimen handling error (sample swapped during testing) at the reference laboratory  ^d^ Target Not Detected  ^e^ Participant had no risk factors  ^f^ Participant is not symptomatic | | | | | | | | | | |
